# Modification of Some Structural and Functional Parameters of Living Culture of *Arthrospira platensis* as the Result of Selenium Nanoparticle Biosynthesis

**DOI:** 10.3390/ma16020852

**Published:** 2023-01-15

**Authors:** Liliana Cepoi, Inga Zinicovscaia, Tatiana Chiriac, Ludmila Rudi, Nikita Yushin, Dmitrii Grozdov, Ion Tasca, Elena Kravchenko, Kirill Tarasov

**Affiliations:** 1 Institute of Microbiology and Biotechnology, Technical University of Moldova, 1 Academiei Str., 2028 Chisinau, Moldova; 2 Joint Institute for Nuclear Research, 6 Joliot-Curie Str., 141980 Dubna, Russia; 3 Horia Hulubei National Institute for R&D in Physics and Nuclear Engineering, 30 Reactorului Str. MG-6, 077125 Bucharest, Romania; 4 Institute of Chemistry, 3 Academiei Str., 2028 Chisinau, Moldova; 5 Doctoral School of Biological, Geonomic, Chemical and Technological Science, State University of Moldova, 2009 Chisinau, Moldova

**Keywords:** selenium nanoparticles, spirulina, bioaccumulation, proteins, genes

## Abstract

Selenium nanoparticles are attracting the attention of researchers due to their multiple applications, including medicine. The biosynthesis of selenium nanoparticles has become particularly important due to the environmentally friendly character of the process and special properties of the obtained particles. The possibility of performing the biosynthesis of selenium nanoparticles via the living culture of *Arthrospira platensis* starting from sodium selenite was studied. The bioaccumulation capacity of the culture, along with changes in the main biochemical parameters of the biomass, the ultrastructural changes in the cells during biosynthesis and the change in the expression of some genes involved in stress response reactions were determined. Protein, lipid and polysaccharide fractions were obtained from the biomass grown in the presence of sodium selenite. The formation of selenium nanoparticles in the protein fraction was demonstrated. Thus, *Arthrospira platensis* culture can be considered a suitable matrix for the biosynthesis of selenium nanoparticles.

## 1. Introduction

Selenium is an indispensable microelement for life, being a component in antioxidant enzymes. Its deficiency can lead to the development of cancer or cardiovascular diseases, whilst an excess of selenium is extremely toxic to living cells. Thus, the use of selenium in different treatments requires a very careful approach, due to the very narrow range between therapeutic and toxic doses [1].

Nanoparticles are particles with diverse properties and a high level of activity compared to bulk elements. In this regard, selenium nanoparticles (SeNPs) are very attractive as alternative remedies for various applications. Thus, SeNPs produced in different ways can be considered a good alternative to antibiotics or preparations designed for cancer treatment [2,3,4,5]. For example, SeNPs obtained by pulsed laser ablation in a liquid method showed that antibacterial action against standard and antibiotic-resistant phenotypes of Gram-negative and Gram-positive bacteria was not toxic to healthy skin tissues, but had anticancer effects on human melanoma and glioblastoma cells at a concentration of 1 ppm [3]. In addition, SeNPs have antioxidant properties, as well as anti-inflammatory and anti-diabetic action [1]. In recent years, attention has also been drawn to the theranostic potential of SeNPs [6].

In contrast to other types of nanoparticles, SeNPs are of great prospects for the treatment of cardiovascular diseases. SeNPs, in addition to replacement of the selenium-deficiency characteristic for heart patients, also have the ability to transfer electrical signals in seeded scaffolds [7]. A recent study showed that SeNPs modify chitosan used as a biofilm-forming material in cardiac surgery so that it acquires electrical conductivity that ensures rapid coupling between cardiomyocytes, which significantly improves the mechanical properties of the myocardium [8]. Other nanoparticles with such properties, for example gold ones, are characterized by pronounced cardiotoxicity [9]. SeNPs inhibit the formation of atherosclerosis in apolipoprotein-E-deficient mice by inhibiting hyperlipidemia through suppressing hepatic cholesterol and fatty acid metabolism and alleviate oxidative stress, thereby enhancing antioxidant activity [10]. These properties in SeNPs are explained by their higher biocompatibility compared to other type of nanoparticles, which is ensured by the fact that they can be formed in the cytosol of cells, where elemental selenium resulting from the metabolism of selenites is accumulated [11].

Currently, SeNPs are produced using physical, chemical and biological methods. Biological methods of nanoparticle synthesis are becoming more and more attractive due to the low toxic effects on the environment and low production price [12]. SeNP biosynthesis can be achieved in two ways: using living cells or using different extracts obtained from cells. In both cases, formation of nanoparticles is based on the reduction potential of the matrix used for the synthesis. In the case of living organisms, the reduction of selenite and selenate ions to SeNPs is seen as a strategy to reduce the toxicity of inorganic selenium.

The most frequently used living matrices for the biosynthesis of SeNPs are fungal cultures [13,14,15,16] and bacteria [12,17,18,19]. Cyanobacteria also present great interest for the biosynthesis of SeNPs. Thus, the biosynthesis of SeNPs by *Hapalosiphon* sp. [20], *Synechocystis* sp. [21], *Microcystis aeruginosa* [22], *Anabaena* sp. [23], *Anabaena variabilis*, *Arthrospira indica*, *Gloeocapsa gelatinosa*, *Oscillatoria* sp. and *Phormidium* sp [24] was reported.

Spirulina (*Arthrospira platensis*), due to its unique therapeutic properties, has also been studied as a matrix for the biosynthesis of SeNPs. However, often, the conditions under which these nanoparticles are produced are incompatible with the normal vital processes for cyanobacteria, which are subject to pronounced biodegradation processes. For example, in a previously performed study, to obtain SeNPs, spirulina separated from cultivation medium was transferred to sodium selenite solution, which does not contain the necessary elements to ensure the vital processes of spirulina, as well as the optimal level of salinity and pH not respected. As a result, the process of SeNP biosynthesis was associated with severe biomass biodegradation [25]. A successful synthesis of SeNPs by spirulina (*Spirulina platensis*) was achieved at pH 7.0 when applying a 12:12 photoperiodic regime, as was reported by Alipour and co-authors [26]. The authors obtained homogeneous SeNPs with antioxidant properties, significantly exceeding those of the sodium selenite solution. A current review reporting the research in the field of nanoparticle biosynthesis highlighted their biosynthesis by different cyanobacteria, in particular, extracellularly or on the surface of the cell wall [27]. The intracellular synthesis of SeNPs takes place due to the activity of two main enzymes—selenate reductase and selenite reductase—while extracellular synthesis occurs under the action of different organic compounds with reducing potential [27].

At the same time, different changes in the cyanobacteria cells subjected to selenium ions were mentioned. They include inhibition or reduction in the culture growth rate at certain element concentrations, modification of biochemical parameters, antioxidant properties, the level of reactive oxygen species in cells, structural changes in cells and modification of the expression of some genes associated with the photosynthesis process [20,21,22,23,28,29].

Obtaining spirulina (*Arthrospira platensis*) biomass, known for its multiple therapeutic benefits, supplemented with biogenic SeNPs seems to be a very interesting direction, both in terms of fundamental and applied research. The main goal of the present study was to obtain selenium nanoparticles using *Arthrospira platensis*, by adding selenium salt during biomass growth, maintaining high biomass productivity.

## 2. Materials and Methods

### 2.1. Experiment Design

As the object of the study, cyanobacteria *Arthospira platensis* (*A. platensis*, spirulina) CNMN-CB-02 from collection of non-pathogenic microorganisms (Institute Microbiology and Biotechnology, TUM, Chisinau, Moldova) was used. The composition of the medium used for biomass growth is presented in [30]. The amount of inoculum was 0.4–0.45 g/L. The culture was cultivated at temperature 28–32 °C, pH—8–10 and continuous illumination with an intensity of 55–85 µM photons/m^2^/s. The culture was shaken daily for 2 h. The duration of the cultivation cycle was 6 days. On the third day of the cultivation cycle, Na_2_SeO_3_ in concentrations 25–200 mg/L, with a step of 25 mg/L, was added to the spirulina medium. At the end of the cultivation cycle, biomass was separated from the medium by filtration. Selenium concentration in initial and experimental solutions was determined using an inductively coupled plasma-optical emission spectrometer, PlasmaQuant 9000 Elite (Analytik Jena, Jena, Germany). Calibration solutions and standards for measurements were prepared from IV-STOCK-27 (Inorganic Ventures, Christiansburg, VA, USA) standard solution. All control standards were analyzed after every 5 samples. For the biochemical tests, the biomass was subjected to a repeated freezing–thawing procedure.

### 2.2. Determination of Selenium Content in Biomass

The determination of selenium content in spirulina biomass and its fractions was carried out according to GOST R 51309-99 “Drinking water. Determination of elements content by atomic spectrometry methods”, which is based on the vaporization of the solution to be analyzed containing selenium in an air flame with acetylene and the measurement of the absorbance of the flame (vapors containing selenium) at wavelengths 196–207.5 nm. The determinations were made based on the calibration curve. Biomass mineralization was primarily produced by using concentrated H_2_O_2_ and HNO_3_. The biomass was mineralized for 2–3 h in a sand bath until a colorless solution was obtained.

### 2.3. Determination of the Amount of Spirulina Biomass

The amount of biomass was determined spectrophotometrically by recording the absorbance of the suspension at a wavelength of 750 nm and the subsequent calculation in g/L.

### 2.4. Determination of the Biochemical Composition of Spirulina Biomass

The content of protein was determined based on the principle of formation of copper complex with peptide bonds and its subsequent reduction under alkaline conditions. The formed complex reduces the Folin-Ciocalteu reagent (Lowry Method). The content of carbohydrates was determined spectrophotometrically based on the formation of hydroxymethylfurfural under interaction of carbohydrates with the Anthon reagent (C_14_H_10_O) in acid medium. For determination of phycobiliprotein content, we used the water extract obtained by repeated freezing–thawing of 10 mg of biomass in 1 mL of distillated water. The absorbance of the supernatant was measured at 620 nm for c-phycocyanin and 650 nm for allophycocyanin. The lipid content was determined spectrophotometrically using phosphovanilinic reagent. Malondialdehyde (MDA) was determined based on reactive products of thiobarbituric acid. The content of chlorophyll a and β carotene was determined spectrophotometrically based on the determination of absorbance of ethanolic extract obtained from spirulina biomass. More detail about each parameter determination can be found in [30].

### 2.5. RNA Extraction and Quantitative RT-PCR (RT-qPCR)

Total RNA was extracted from the pellet obtained after centrifugation of 25 mL of algal culture in three biological repeats using TRIzol™ Reagent (Invitrogen™, Waltham, MA, USA) according to manufacturer’s protocol. The integrity of isolated RNA was evaluated using an QIAxcel Advanced system (Qiagen, Hilden, Germany). cDNA was obtained with Maxima First Strand cDNA Synthesis Kit for RT-qPCR, with dsDNase (Thermo Scientific™, Waltham, MA, USA) following the manufacturer’s instructions. The RT-qPCR was performed using iTaq Universal SYBR Green Supermix (BioRad, Hercules, CA, USA) on a CFX96 Touch Real-Time PCR Detection System (BioRad, USA) with primers for the following genes: FeSOD, GOGAT, hsp90, rbcL, POD and 16S rRNA (reference) (Table S1). The relative transcript levels of the selected genes were analyzed using the ΔΔCt method.

### 2.6. Transmission Electron Microscopy (TEM) Analysis and Energy Dispersive X-ray Analysis

The morphology of spirulina cells (control and exposed to selenium) was described using a JEM-1400 transmission electron microscope (Jeol, Akishima, Japan) at an accelerating voltage of 100 kW. Microprobe analysis of SeNPs was conducted with an energy-dispersive X-ray analysis spectrometer (EDAX, Pleasanton, CA, USA). The acquisition time ranged from 60 to 100 s, and the accelerating voltage was 20 kV.

## 3. Results and Discussion

### 3.1. Uptake of Se and Its Accumulation in the Arthrospira platensis Biomass

**Figure 1:**
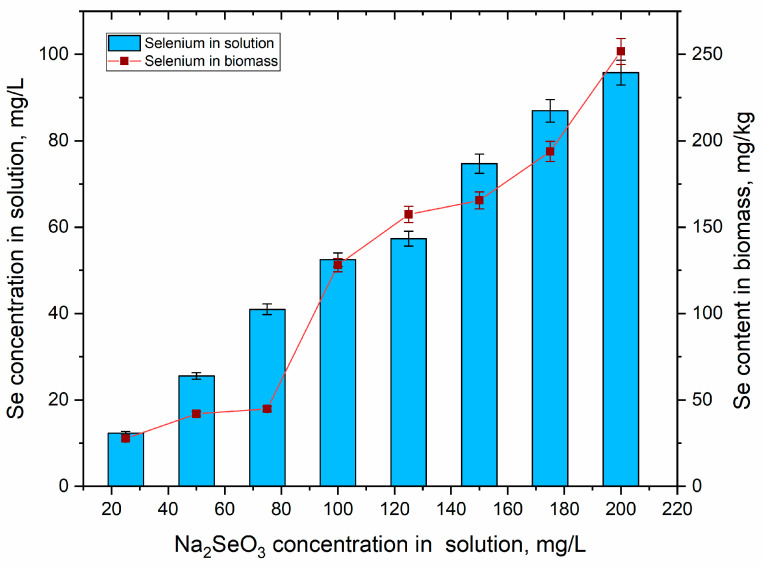
Selenium accumulation in *Arthrospira platensis* biomass (n = 3, the error bars represent the standard deviation of the measurements).

The removal of selenium by *A. platensis* from the nutrient medium varied between 12.8 and 26.6% (Figure 1). The lowest Se removal from medium was attained at a Na_2_SeO_3_ concentration of 75 mg/L (the selenium concentration being 47.03 mg/L), while the highest at a Na_2_SeO_3_ concentration of 125 mg/L (the selenium concentration being 78.38 mg/L).

Selenium content in biomass increased with the rise in its concentration in the nutrient medium. At a Na_2_SeO_3_ concentration range of 25–75 mg/L, the content of selenium in biomass varied between 28 and 45 µg/g. At a Na_2_SeO_3_ concentration of 100 mg/L, the content of selenium in the biomass was 128 µg/g, which is 2.9-times higher compared to the value obtained at a salt concentration of 75 mg/L. A further increase in the Na_2_SeO_3_ concentration in the spirulina cultivation medium resulted in a slow continuous accumulation of selenium in biomass, reaching a value of 250 µg/g at a salt concentration of 200 mg/L.

It is known that spirulina efficiently accumulates selenium from various salts, including sodium selenite, in a dose- and time-dependent manner [31,32]. The tolerance of cyanobacteria to selenite is relatively high. For example, different strains of spirulina growth were not inhibited by sodium selenite present in the medium in concentrations up to 400 mg/L [32]. Selenium accumulation in biomass can also be influenced by other factors, such as sulfur concentration in the medium or the presence of various organic compounds [22,31]. In the present study, spirulina was grown at optimal conditions, ensuring the accumulation of a large amount of high-quality biomass; thus, a balance can be obtained between all the valuable components in the biomass, including the amount of accumulated selenium. Since high concentrations of Na_2_SeO_3_ led to unfavorable changes in biomass composition, the effect of concentrations higher than 200 mg/L was not analyzed, even though they may ensure a higher level of accumulation of selenium in biomass.

### 3.2. The Content of Selenium in Arthrospira platensis Biomass Fractions

**Figure 2:**
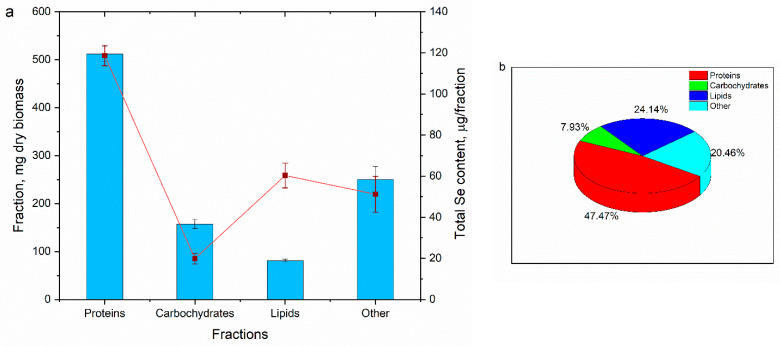
Amount of Se accumulated in biomass fractions: (**a**) total content of Se, µg Se/g per fraction; (**b**) distribution of Se accumulated by fractions, % of the accumulated amount (n = 3, the error bars represent the standard deviation of the measurements).

In order to identify the cellular components mainly responsible for selenium accumulation, the biomass grown in the presence of Na_2_SeO_3_ at a concentration of 200 mg/L was subjected to fractionation, after which the selenium content in each fraction was determined. The results are shown in Figure 2.

The highest content of selenium was determined in the protein fraction, constituting 118.7 µg relative to the amount of the extracted protein, so an accumulation capacity of 0.23 µgSe/mg of proteins was obtained. The amount of lipids extracted from spirulina biomass amounted to 81 mg/g of dry biomass. In this fraction, 60.34 µg of Se was accumulated, which is the second-highest amount of accumulated selenium in terms of absolute values. The capacity of selenium accumulation by lipids was significantly higher compared to proteins and constituted 0.74 µgSe per mg of extracted lipids. Carbohydrates showed the lowest selenium accumulation capacity, in absolute numbers, accumulating 19.8 µg in the integral fraction of carbohydrates extracted from one gram of biomass, and in relative values, 0.126 µgSe per mg of extracted carbohydrates. Thus, almost half of the selenium (47.47%) was accumulated in the protein fraction, 24.14% in the lipid fraction and 7.93 in the carbohydrate fraction.

A similar distribution of selenium accumulation by spirulina was reported in other studies. For example, the same pattern was noticed when spirulina accumulated selenium from other selenium compounds, such as iron selenite hexahydrate or germanium selenide [29]. Li and co-authors demonstrated that over 85% of the selenium accumulated in spirulina biomass from inorganic salts is converted to organic selenium. Approximately one-quarter of the total selenium was accumulated in the polypeptide fraction, more than 10% was accumulated in lipids and approximately 2% in carbohydrates. It was assumed that approximately half of the accumulated selenium bound to free amino acids, oligopeptides or other similar compounds [32].

### 3.3. Formation of Selenium Nanoparticles

**Figure 3:**
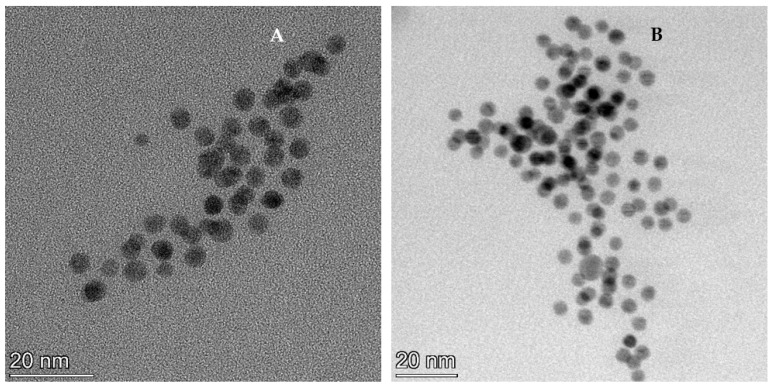

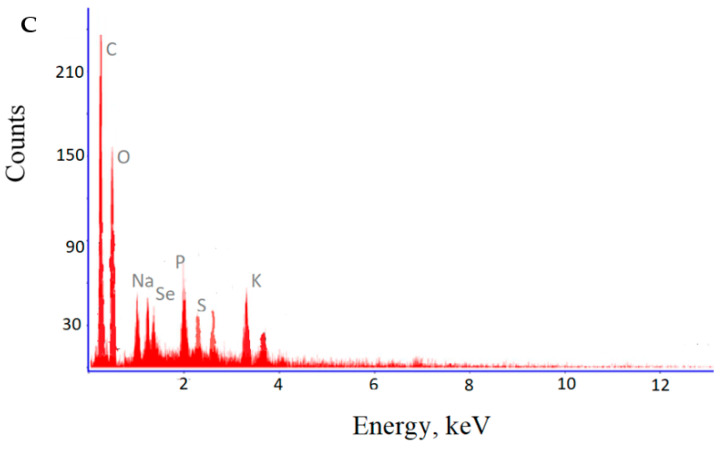
SeNPs in protein fraction ((**A**)—camera imaging 634.1 kx Ceta; (**B**)—STEM diffraction 802.3 kx BF, (**C**)—EDX spectra of selenium nanoparticles).

The obtained fractions of biomass were subjected to microscopic examination (TEM) in order to identify SeNPs, in case of their formation. Nanostructures were identified only in the total protein fraction (Figure 3). From the obtained images, it can be seen that SeNPs formed in the protein fractions were scattered and spherical, with a size in a range of 2–8 nm (Figure 3). The size of nanoparticles, including selenium, determines both their ability to reach target tissues as well as their retention capacity. Small-sized nanoparticles exhibit significant tumor targeting, with minimum to no nonspecific uptakes, but are also characterized by faster elimination [33]. A final hydrodynamic diameter of nanoparticles smaller than 5.5 nm resulted in their rapid and efficient renal excretion and elimination from the body [34,35]. However, recently, this statement has been questioned, since even nanoparticles of larger sizes but with specific properties are easily eliminated through the kidney [36]. Nevertheless, small nanoparticles are quickly eliminated from the bloodstream through the kidneys. This is of major importance when SeNPs are applied in cancer treatment—nanoparticles that have not been accumulated by tumor tissues are quickly removed from the bloodstream, thereby preventing undesirable effects on other tissues and organs.

According to the previously published results, the formation of nanoparticles by spirulina culture takes place preferentially extracellularly [27]. At the same time, there is evidence that prokaryotic organisms can produce SeNPs intracellularly, this being an active process involving the specific enzymes selenate reductase and selenite reductase. Selenite can be also reduced to elemental Se in a non-enzymatic way with the participation of glutathione (GSH) or enzymatically using nonspecific enzymes, such as respiratory and/or detoxifying enzymes, for example, periplasmic nitrite reductase and sulfite reductase [37].

It is known that at high concentrations of selenium, in the present study, 200 mg/L, *A. platensis* is able to resist the action of selenite ions through their reduction to elemental selenium. Selenium is poorly soluble and, therefore, less toxic than selenite ions [32]. Thus, the cyanobacteria apply, in this case, the tactics of reducing toxicity, ensuring a fairly high degree of tolerance to this element.

Depending on the biosynthesis conditions, as, for example, the precursor used and the microorganism culture used as a matrix, SeNPs with a wide range of sizes (from 5 to 530 nm) can be obtained [4,17,18,19,26]. Nanoparticles obtained intracellularly are smaller compared to those produced extracellularly. In the present study, the size of the nanoparticles was closer to the minimum size limit reported in the literature.

The presented results were obtained under laboratory conditions, when small volumes were used, and their extrapolation to large-scale production requires further appropriate experiments. However, starting from the fact that performing a biosynthesis process at conditions optimal for spirulina growth allows for maintaining a high level of biomass productivity, it can be assumed that the success of this method transfers to the industrial level.

### 3.4. Content of Biomass and Its Biochemical Composition

**Figure 4:**
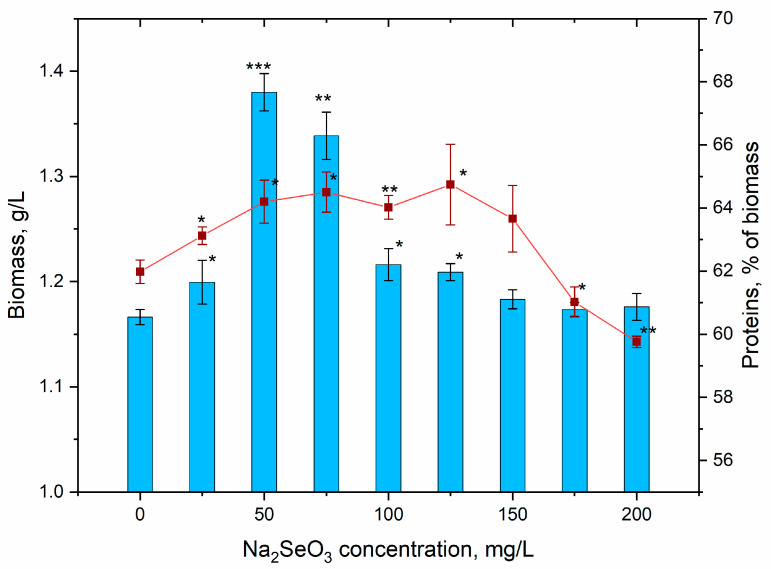
Accumulation of *Arthrospira platensis* biomass and content of protein in biomass under conditions of supplementing the nutrient medium with sodium selenite (* *p* < 0.05, ** *p* < 0.005, *** *p* < 0.0005 for differences between control and experimental sample; n = 3). The error bars represent the standard deviation of the measurements.

The accumulation of *A. platensis* biomass at its cultivation in standard conditions and in the presence of different concentrations of sodium selenite can be seen in Figure 4.

The applied concentrations of Na_2_SeO_3_ did not have a pronounced inhibiting effect on the biomass accumulation during spirulina growth. At salt concentrations of 150, 175 and 200 mg/L, the differences in biomass accumulation in experimental and control samples were not statistically significant. At other Na_2_SeO_3_ concentrations, a statistically significant increase in the biomass took place. However, in absolute values, the increase in the amount of biomass was important only at Na_2_SeO_3_ concentrations of 50 and 75 mg/L, when the amount of biomass increased by 18.4 and 14.8%, respectively, compared to the control. The stimulatory effects of low concentrations of selenium present in the medium on spirulina growth were obtained when other selenium compounds were used or other experimental conditions were applied [23,24,28]. It is considered that an external supply of Se (by element) up to 150 mg/L has beneficial effects on the accumulation of spirulina biomass [38]; however, it should be mentioned that there is a pronounced dependence between the level of spirulina tolerance to selenium and the experimental conditions.

The stimulating effects of selenium on biomass productivity were observed not only for spirulina, but for other cyanobacteria as well. For example, the growth of the cyanobacterium *Hapalosiphon* sp. was stimulated by Se at concentrations up to 200 ppm, while higher concentrations inhibited biomass growth [20]. In the cyanobacteria *Synechocystis* sp., selenium concentrations up to 200 ppm of selenite did not provide an increase in the amount of biomass, but neither did they inhibit it [21].

The protein content in the biomass of spirulina grown in the presence of different concentrations of sodium selenite varied within very narrow limits and constituted 59.76–64.74% of the dry biomass (Figure 4). The control biomass contained 61.98% of proteins. Although the limits of variation were small, the observed differences have statistical significance, and it should be mentioned that in the case of salt concentrations of 25–125 mg/L, a slight increase in the content of proteins was observed. At a Na_2_SeO_3_ concentration of 150 mg/L, the content of protein in biomass did not differ from the control, while at concentrations of 175 and 200 mg/L, a slight decrease was observed.

**Figure 5:**
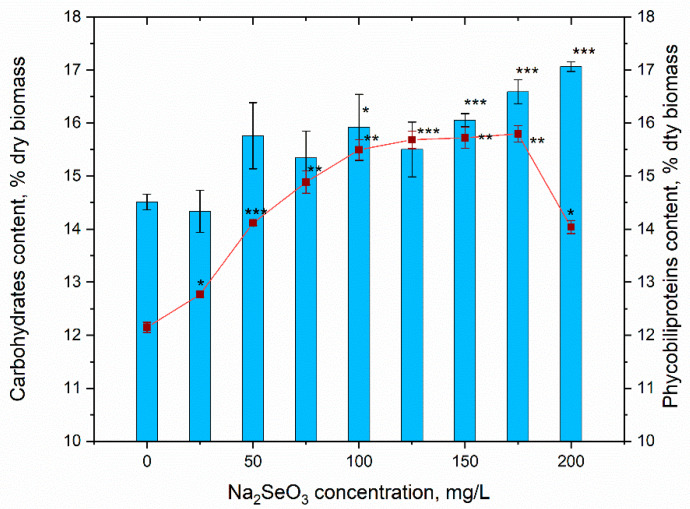
The content of carbohydrates and phycobiliptoteins in *Arthrospira platensis* biomass under conditions of supplementing the nutrient medium with sodium selenite (* *p* < 0.05, ** *p* < 0.005, *** *p* < 0.0005 for differences between control and experimental sample; n = 3). The error bars represent the standard deviation of the measurements.

Carbohydrates and phycobiliptroteins are two components in spirulina biomass, which are extremely labile and react to different external factors (Figure 5). In conditions of toxicity of external effects, an increase in the amount of carbohydrates and a concomitant decrease in the content of phycobilins are observed. In the present study, however, a pronounced increase in the content of both groups of compounds, which can be more associated with the effect of stimulating certain biosynthetic processes in the spirulina culture, was observed. The highest increase in carbohydrate content—by 10.6–17.6% compared to the control—was observed at sodium selenite concentrations of 150–200 mg/L. Similarly, at these concentrations, the level of significance of the differences between experimental samples and control was high (*p* < 0.005). In the case of the other concentrations, the differences between control and samples were lower or not observed.

The total content of phycobiliproteins in the spirulina biomass obtained in the experimental variants was significantly higher compared to the control (by 5.1–30.0%). The lowest difference between the control and the experiment samples was observed at a sodium selenite concentration of 25 mg/L. With the increase in the salt concentration, the content of total phycobiliproteins in the biomass also increased. Thus, at a concentration of sodium selenite of 50 mg/L, the amount of phycobiliproteins increased by 16.1% compared to the control, at a concentration of 75 mg/L—by 22.5% and at concentration of 100 mg/L—by 27.5%. An increase in the Na_2_SeO_3_ concentration from 100 to 175 mg/L did not result in a further increase in the content of phycobilins, the amount of these compounds remaining at the plateau level reached at a Na_2_SeO_3_ concentration of 100 mg/L. The Na_2_SeO_3_ concentration of 200 mg/L was characterized by a reduction in the level of phycobiliproteins by 14.6% compared to the concentration of 175 mg/L. Although it is an important decrease, the total content of phycobiliproteins in the spirulina biomass still remained quite high compared to the control, exceeding it by 15.5%.

**Figure 6:**
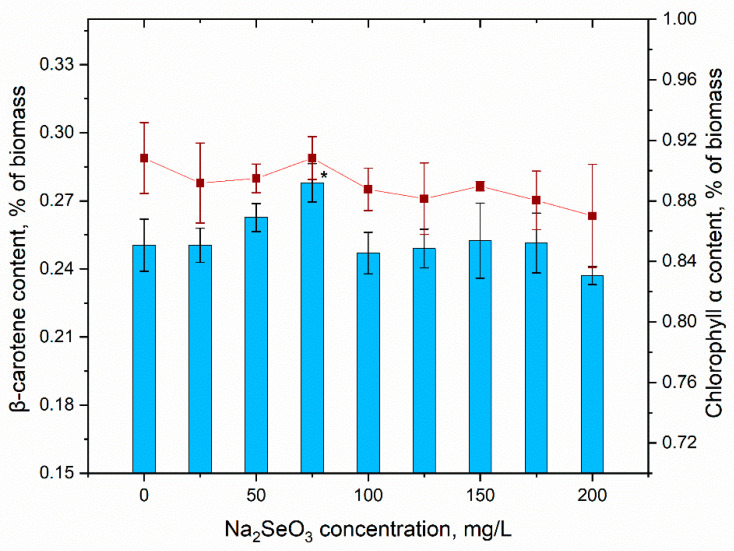
The content of β carotene and chlorophyll a in *Arthrospira platensis* biomass under conditions of supplementing the nutrient medium with sodium selenite (* *p* < 0.05 for differences between control and experimental sample; n = 3). The error bars represent the standard deviation of the measurements.

Primary photosynthetic pigments are most often studied as a response reaction of photosynthetic organisms to the action of various external factors. In this study, the change in the content of chlorophyll α and β carotene in the control and experimental biomass was monitored (Figure 6).

As can be seen from the presented data, the content of chlorophyll α and β carotene did not change, or changed insignificantly in the biomass of spirulina grown on the medium with the addition of sodium selenite at concentrations up to 200 mg/L. Only in the case of a concentration of 75 mg/L, an increase by 11.2% (statistically veridical at a level of significance *p* < 0.05) in the carotene content in the spirulina biomass was observed. For other Na_2_SeO_3_ concentrations, the level of these two extremely important pigments was comparable with control values. To a large extent, the quantitative stability of these cellular components ensures an adequate level of assimilation processes and, therefore, the growth of cyanobacteria under conditions of the presence of selenite ions in the nutrient medium.

**Figure 7:**
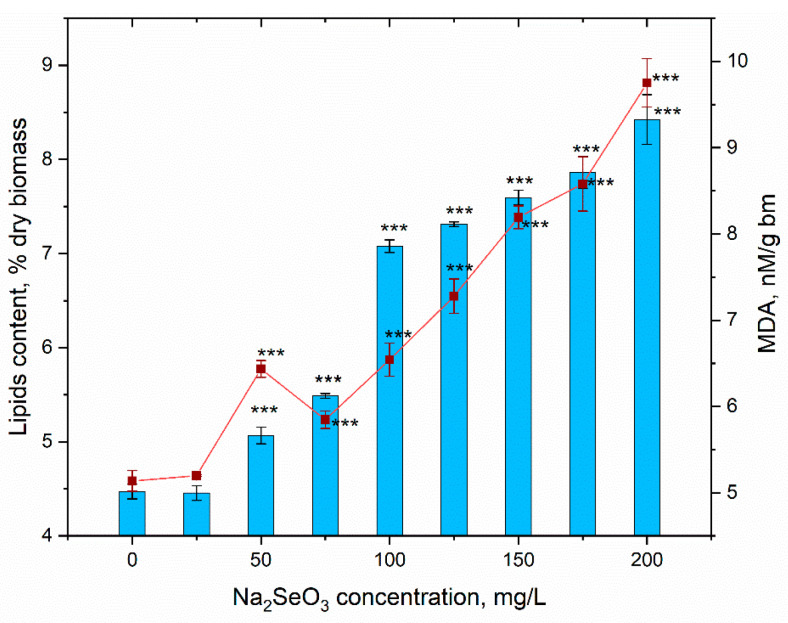
The content of lipids and MDA in *Arthrospira platensis* biomass under conditions of supplementing the nutrient medium with sodium selenite (*** *p* < 0.0005 for differences between control and experimental sample; n = 3). The error bars represent the standard deviation of the measurements.

The greatest quantitative changes in biochemical parameters were observed at the level of lipids and end products of their peroxidation (Figure 7).

Starting from a sodium selenite concentration of 50 mg/L, a significant increase in the lipid content in the spirulina biomass was observed. This increase was in a dose-dependent manner, amplified by a rise in salt concentration in the medium. Thus, at a Na_2_SeO_3_ concentration of 50 mg/L, the content of lipids increased by 13.3% compared to the control; then, at a concentration of 200 mg/L, this increase was 88.5%. Practically the same pattern was observed for the malonic dialdehyde and its content in biomass increased by 13.7–89.7% compared to control. The content of MDA increased significantly in spirulina biomass grown in the medium with the addition of sodium selenite, but this is associated with a proportional increase in the content of total lipids in the biomass. Thus, in relative terms, the amount of lipid degradation products related to the amount of lipids remained a stable parameter.

The quantitative increase in lipid peroxidation products in spirulina cells under the influence of sodium selenite was also attested by other researchers [28], but in the mentioned study, the content of total lipids in the biomass was not monitored. In general, however, an increase in the amount of lipid oxidative degradation products is an indicator of toxic effects and should be treated with caution.

### 3.5. Gene Expression Analysis

**Figure 8:**
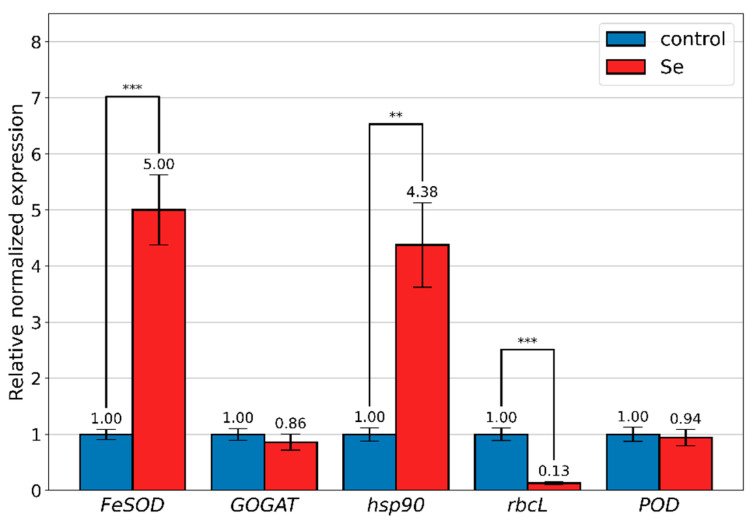
Relative expression difference in selected genes between control *A. platensis* and after Se treatment (200 mg/L of Na_2_SeO_3_), ** *p* < 0.01, *** *p* < 0.001; n = 3. The error bars represent the standard deviation of the measurements.

In the present study, one of the aims was to highlight the change in the expression of some genes associated with the generalized response to stress conditions. Figure 8 shows the results with reference to the change in the expression of *FeSOD*, *hsp90*, *rbcL*, *GOGAT* and *POD* genes in *A. platensis* cells that were grown in the presence of sodium selenite applied at a concentration of 200 mg/L.

To estimate the possible response to selenium treatment, the transcriptional abundance of heat-shock protein (*HSP90*), glutamate synthase (*GOGAT*), iron-superoxide dismutase (*FeSOD*), peroxidase (*POD*) and the large subunit of Rubisco (*rbcL*) genes was measured in the control and *A. platensis* biomass treated with 200 mg/L of sodium selenite. The results showed the increased expression level for *FeSOD* and *hsp90* genes and decreased transcription level for *rbcL* (Figure 8). The expression levels of *GOGAT* and *POD* genes were not significantly different between control cells and cells treated with selenium.

Previous research has shown that cyanobacterial cells can change the expression of a number of genes in response to stressful conditions, such as genes encoding heat-shock proteins or genes involved in metabolic activity and the production of antioxidant enzymes [39,40,41]. In the present study, the expression level of five genes—*FeSOD*, *GOGAT*, *hsp90*, *rbcL* and *POD*—was assessed.

The *FeSOD* and *POD* genes encode two of the most important first-line antioxidant enzymes of spirulina—Fe-component superoxide dismutase and peroxidase. The first enzyme catalyzes the dismutation reaction of the superoxide radical into hydrogen peroxide, and the peroxidase—the decomposition reaction of peroxide into water and oxygen. In addition to the production of antioxidant enzymes, the defense mechanisms of cyanobacteria in relation to stress factors include the inclusion of other genes associated with the stress response, such as the genes of heat-shock proteins (HSPs), ribulose bisophosphate carboxylase/oxygenase (Rubisco), glutamate synthase and GOGAT [42].

The increase in the relative expression of the *FeSOD* gene is predictable under stress conditions. In the present study, stress conditions were not confirmed by means of monitoring changes in biomass production or changes in biochemical parameters. In contrast, a 5-fold increase in *FeSOD* expression compared to the control can be considered as evidence of unfavorable oxidative stress in *A. platensis* cells grown in the presence of 200 mg/L of sodium selenite. The situation may become more complicated over time because the expression level of POD, which is encoding peroxidase, remained at the control level, this enzyme being responsible for removal of the reaction product (H_2_O_2_) formed as a result of superoxide dismutase activity.

HSPs are involved not only in the case of thermal stress, but also in other forms of stress, because they ensure the stability of proteins and the avoidance of their misfolding [42,43,44,45]. In the case of the influence of selenium on *A. platensis,* an increase in the transcriptional abundance of *hsp90* (4.4-times compared to the control) was observed, which points to a need to maintain the stability of cellular proteins. The same upregulation of *hsp90* was observed, for example, when growing spirulina at temperatures lower or higher than the optimum one [42]. The role of HSPs in the adaptation to oxidative stress conditions caused by physical (radiation) and chemical factors (action of methyl viologen) was demonstrated in *Synechococcu* sp. [46].

Gene *rbcL* (the large subunit of ribulose bisphosphate carboxylase/oxygenase; Rubisco) is involved in ensuring the growth of photosynthetic organisms and the metabolic activity necessary to maintain life, as the *GOGAT* gene, which codes the glutamate synthase enzyme [42,43,44,45,46]. *rbcL* is directly involved in carbon assimilation, thus, ensuring the process of photosynthesis and organism growth, and GOGAT catalyzes the reaction of glutamate formation from glutamines and 2-oxoglutarate. No change in the expression level of *GOGAT* was observed, but the reduction in the expression level of *rbcl* was very significant (7.7-times). Downregulation of *rbcl* is observed in cyanobacteria under different stress conditions, for example, under conditions of low temperature or osmotic stress [47].

Thus, the change in the expression of three genes associated with stress from the five studied confirms that the culture of *A. platensis* was exposed to stress caused by the presence of selenium in the nutrient medium, although, apparently, the cyanobacterial culture developed normally.

### 3.6. Ultrastructural Changes in Arthrospira platensis under the Influence of Selenium

**Figure 9:**
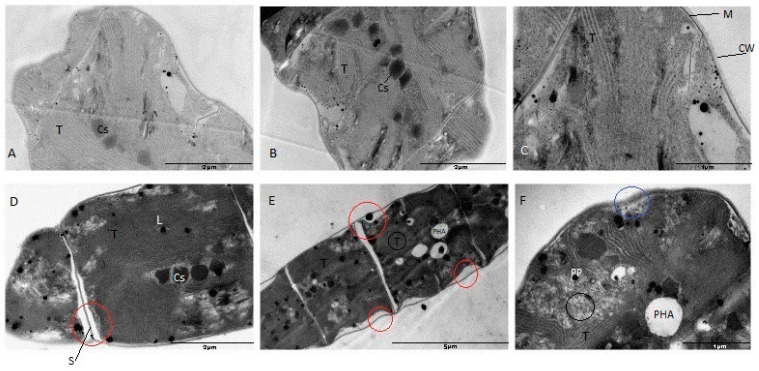
The ultrastructure of *A. platensis* cells (**A**–**C**)—control, (**D**–**F**)—cells grown in the medium containing sodium selenite in concentration of 200 mg/L. Cs—carboxisomes; T—thylacoids; M—membrane; CW—cell wall; L—lipid inclusions; PHA—polyhydroxyalkanoates; PP—polyphosphate bodies; S—septum; black circles—disintegration and disorganization of thylakoids, red circles—space between cell membrane and cell wall/intercellular septum, blue circle—modification of the cell wall/exopolysaccharides density. Scale bar in (**A**) 2 µm, (**B**) 2 µm, (**C**) 1 µm, (**D**) 2 µm, (**E**) 5 µm, (**F**) 1 µm.

The changes that take place in the ultrastructure of *A. platensis* cells can be seen in Figure 9. In Figure 9A–C, the typical spirulina ultrastructure can be seen. The most significant structures characteristic of the cell cytoplasm are compactly arranged thylakoids, well-structured thylakoids with well-visualized membranes. The cytoplasm is dense and adheres tightly to the cytoplasmic membrane. The cell wall is tightly attached to the cytoplasmic membrane; it is dense and well visible.

Under the action of selenium, changes in the structure of spirulina occurred, the main ones being a decrease in the degree of compaction of thylakoids, appearance of translucent spaces between the cytoplasmic membrane and the cell wall, appearance of large polyphosphate bodies, polyhydroxyalkanoates (which are a form of storage of reserves of carbon) and a decompaction of the cell wall.

Such changes are typical for *A. platensis* cells exposed to various external actions and stress factors. For example, in the case of the action of heavy metals (Cd), first of all, the disintegration and disorganization of thylakoid membranes, as well as the appearance of polyphosphate bodies [48] are observed. Destruction of photosynthetic membranes and storage of carbon reserves in the form of polyhydroxyalkanoates was also observed in spirulina under conditions of insufficient nutrients, especially nitrogen [49].

## 4. Conclusions

*Arthrospira platensis* easily tolerates the presence of the high concentrations of selenium (up to 125 mg/L) in the medium, growth and biomass accumulation, being within the limits of the values characteristic for control biomass. Moreover, at selenium concentrations up to 50 mg/L, the amount of biomass accumulated during the cultivation cycle increased by up to 18% compared to the control. Under these conditions, most of the biochemical parameters remained within the normal limits for the species, although the values obtained in the experimental variants may differ significantly compared to the control. The content of the primary photosynthetic pigment did not change significantly, regardless of the selenium concentration applied. The content of lipids and carbohydrates in biomass increased with increasing sodium selenite concentration added to the nutrient medium. The content of protein and phycobilin also increased, and the dose-dependent character of this relationship was maintained up to a concentration of sodium selenite of 175 mg/L. With an increase in the content of lipids, the level of malonic dialdehyde in the cells also increased.

During the cultivation of spirulina in the nutrient media containing sodium selenite, the culture actively accumulates selenium in a dose-dependent manner. Most of the bioaccumulated selenium was determined in the protein (47.5% of the accumulated selenium) and the lipid (24.1%) fractions. In the protein fraction, selenium nanoparticles with a size of 2–8 nm were formed.

As a result of the action of selenium on *Arthrospira platensis*, the expression of genes associated with stress changed. Thus, the expression level of *FeSOD* and *Hsp90* increased, which may be associated with the need to manage the increased flow of reactive oxygen species and to stabilize the proteins subjected to the action of the xenobiotics. The significant decrease in the level of *rbcl* expression could cause a decrease in the efficiency of carbon fixation from carbon dioxide.

Selenium also caused ultrastructural changes in *Arthrospira platensis* expressed in the damage and disorder of thylakoids, the detachment of the cytoplasmic membrane from the cell wall, the change in the density of the cell wall and the formation of carbon reserves in the cells, indicating negative effects of selenium ions.

Thus, *Arthrospira platensis* tolerates high concentrations of selenium, accumulates important amounts of this element and carries out the biosynthesis of selenium nanoparticles, which are mainly located in the protein and lipid fractions. The process is accompanied by biochemical, ultrastructural and gene expression changes associated with the response of spirulina to stress conditions.

## Data Availability

Not applicable.

## Supplementary Materials

The following supporting information can be downloaded at: https://www.mdpi.com/article/10.3390/ma16020852/s1, Table S1: List of primers.
