# Supplementary material for: Modification of Some Structural and Functional Parameters of Living Culture of Arthrospira platensis as the Result of Selenium Nanoparticle Biosynthesis
Source: Materials (Basel). 2023 Jan 15;16(2):852. doi: 10.3390/ma16020852 (PMC9860699; doi:10.3390/ma16020852)
Supplement: Supplementary file 1 [file materials-16-00852-s001.zip › materials-2139662-supplementary.pdf]

Table S1. List of primers

Gene	Primer sequence
rbcL	CCTGTTGCTTCCGGTGGTAT<br>CACACGGTTAGCAGTTGCAC
GOGAT	TCCTCCTCTTCCTGACCTGG<br>CCGCCAGCGGAATATCATCT
FeSOD	GAAGTGCCTCAAGCCCAATG<br>GCCCAACCACTACCGAACTG
hsp90	CAATTACACCCCGCAAAGGC<br>CCAAGAAGAACCCCTGCTGT
Pod	CCGGATCTTTGAGGGAGGGA<br>TTGCGGGTGATTGACTCCTT
16S rRNA	CGTAAACCTCTCCTCAGTTCAG<br>GAACGGATTCACCGCAGTAT
